# Changes of Bone-Related Minerals during Denosumab Administration in Post-Menopausal Osteoporotic Patients

**DOI:** 10.3390/nu9080871

**Published:** 2017-08-13

**Authors:** Takako Suzuki, Yukio Nakamura, Hiroyuki Kato

**Affiliations:** 1 Department of Orthopedic Surgery, Shinshu University School of Medicine, Asahi 3-1-1, Matsumoto 390-8621, Japan; takako1119@shinshu-u.ac.jp (T.S.); hirokato@shinshu-u.ac.jp (H.K.); 2 Department of Orthopedic Surgery, Showa-Inan General Hospital, Akaho 3230, Komagane 399-4117, Japan

**Keywords:** bone mineral density, bone-related minerals, denosumab, osteoporosis

## Abstract

Objectives: This retrospective study included 21 patients with primary osteoporosis who were treated with the anti-resorption drug, denosumab. To date, there has been no detailed report on the changes of bone-related minerals after anti-resorption drug therapy. Methods: Twenty-one post-menopausal females were retrospectively enrolled. Serum zinc (Zn), magnesium (Mg), iron (Fe), copper (Cu), grip strength, and estimated glomerular filtration rate (eGFR) were examined at one week and 1, 2, 4, 6, 8, 10, and 12 months. Lumbar spine (L1-4) bone mineral density (L-BMD) and bilateral total hip BMD (H-BMD) were examined before and at 4, 8, and 12 months after treatment commencement. Results: Serum Zn tended to decrease at one week and one month, and tended to increase during 10 to 12 months. Serum Cu maintained during zero to eight months, then decreased at 10 and 12 months. Serum Fe gradually increased after four months. Serum Mg sharply increased at one week, then decreased further. Grip strength increased for two months, then slightly decreased and maintained 4 to 12 months. eGFR almost maintained for zero to eight months, then slightly decreased thereafter. L-BMD values significantly increased at eight (5.8%) (*p* < 0.01) and 12 months (9.8%) (*p* < 0.01). H-BMD increased during the period (at 12 months: 3.7%). Conclusions: These results suggest that at later phases of denosumab therapy, Zn and Fe tended to increase while Mg tended to decrease, all of which are important for bone metabolism. Thus, denosumab might improve Zn and Fe metabolism, and thereby likely increase BMD. Since denosumab may not improve Mg, it is better to obtain Mg supplementation during the therapy.

## 1. Introduction

Osteoporosis (OP) is the most common metabolic bone disorder worldwide, and is a major public health concern among the elderly. It is therefore of global importance to reduce the burden of this debilitating disease. The pathogenesis of osteoporosis is complex and multi-factorial. On the other hand, bone minerals, hormone changes, and other factors are probably relevant pathogenic factors in the disease [1].

All currently-approved therapies for osteoporosis involve the use of anti-resorption agents to act on osteoclasts to prevent further bone loss. Denosumab is a fully-human monoclonal antibody that inhibits receptor activator of nuclear factor kappa-B ligand (RANKL), which selectively inhibits osteoclastogenesis. Recent literature on denosumab showed that the drug progressively and linearly increased bone mineral density (BMD) in the spine over an eight-year period and increased total hip and femoral neck BMD to a greater extent during the first three years of treatment than during years 4 to 6 [2,3]. However, it is largely unknown how anti-resorption drugs including denosumab affect bone mineral metabolism in post-menopausal women.

There have been some reports that denosumab affects calcium metabolism (e.g., hypocalcemia) [4,5]. In the Denosumab Fracture Intervention Randomized Placebo Controlled Trial carried out in Japan, Sugimoto et al. reported that all patients who took daily supplements containing ≥600 mg calcium and 400 IU vitamin D had a significantly decreased risk of vertebral fracture and no hypocalcemia when taking denosumab for three years [6]. Thus, it is considered that denosumab might have effects on bone-related minerals during the treatment; however, such data do not exist to date.

Minerals such as magnesium (Mg), zinc (Zn), copper (Cu), and iron (Fe) are all essential for health, and play a role in the synthesis of collagen and other proteins that form the structure of bone. Previous reports from the last three decades have shown that Mg and Zn are essential for organic bone matrix synthesis [7], and Mg deficiency may affect the quality of bone by decreasing bone formation, preventing the optimal crystal formation and having a negative effect on parathyroid hormone [8]. Zn has been demonstrated to play a physiological role in the mineralization of bone tissue [9,10]. In their study, Hill et al. showed a relationship between Zn and bone turnover state in elderly adults [11]. The physiological role of Cu in bone metabolism and homeostasis has been largely unknown; however, several studies demonstrated that in elastin- and collagen-containing tissues such as blood vessels, tendon, and bone, there was decreased mechanical strength of these tissues with Cu deficiency [12,13]. Furthermore, due to the mineral deficiency in post-menopausal women with low BMD and the key role of minerals on bone health, supplementation with Mg, Zn, Fe, and perhaps Cu is recommended. Thus, those minerals are essential for our health and bone metabolism. However, there have been no reports on the changes of minerals and BMD after denosumab treatment in post-menopausal osteoporotic women to date.

In this study, we examined the changes of bone-related minerals and BMD in post-menopausal osteoporotic women after denosumab treatment.

## 2. Materials and Methods

### Patient Characteristics

We collected data for 21 post-menopausal female patients who received denosumab treatment between April 2016 and June 2017. The average age was 74.1 ± 2.5 years, and the body mass index (BMI) was 20.3 ± 0.5 (kg/m^2^). Among them, 10 patients had taken bisphosphonates (BPs) (alendronate: 6 cases; risedronate: 2 cases; ibandronate: 2 cases) combined with alfacalcidol (ALF). The average period of BP therapy was 4.2 ± 1.2 years. Eleven patients had taken teriparatide (PTH) combined with ALF.

The inclusion criteria for the study were post-menopausal osteoporotic patients. The exclusion criteria were patients with chronic renal failure (estimated glomerular filtration rate < 40 mL/min/1.73 m^2^), bone metabolic disorder or diabetes mellitus, which affect OP, and fracture within 1 year prior to the study. The diagnosis of primary OP was made in accordance with the revised criteria established by the Japanese Society of Bone and Mineral Research [14].

Serum Zn (reference value: 65–110 μg/dL) by atomic absorption spectrometry, Mg (reference value: 1.8–2.6 mg/dL) by the xylidyl blue method, Fe (reference value: 48–154 μg/dL) by 2-nitroso-5-[*N*-*n*-propyl-*N*-(3-sulfopropyl) amino] phenol method, and Cu (reference value: 68–128 μg/dL) by the colorimetric method were examined at one week and 1, 2, 4, 6, 8, 10, and 12 months. Serum Zn, Mg, Fe, and Cu values were further analyzed after classifying the following two groups: the 11 PTH patients (PTH group) and 10 patients without PTH (BP group).

The estimated glomerular filtration rate (eGFR) (mL/min/1.73 m^2^) was measured. Grip strength was measured three times in both hands using a Jamar hydraulic hand dynamometer (Sammons Preston, Bolingbrook, IL, USA), and the average value of the stronger side was used.

The percentage changes in bone turnover markers and BMD were determined for each time point and compared between the groups by statistical analysis. BMD was measured using a dual-energy X-ray absorption (DXA) fan-beam bone densitometer (Lunar Prodigy; GE Healthcare Bio-Sciences Corp., Piscataway, NJ, USA) at the L1 to L4 levels of the posteroanterior spine (L-BMD) and bilateral hips (H-BMD). H-BMD was calculated as the average BMD of the right and left hips. BMD was examined before treatment administration and at 4, 8, and 12 months.

Results are expressed as the mean ± standard error of the mean. For both groups, we compared the changes in markers, L-BMD, and H-BMD at each time point using the Bonferroni correction method for multiple comparisons. Comparisons of markers, L-BMD, and H-BMD between the test groups at each measurement point were performed using Welch’s *t*-test. Differences were considered statistically significant at *p <* 0.05.

The study protocol was approved by the ethics committees of Shinshu University School of Medicine (Matsumoto, Japan) and Showa-Inan General Hospital (Komagane, Japan). This investigation was carried out in accordance with the ethical standards set forth in the Declaration of Helsinki (2014 revision). Written informed consent was obtained from all patients.

## 3. Results

**Figure 1:**
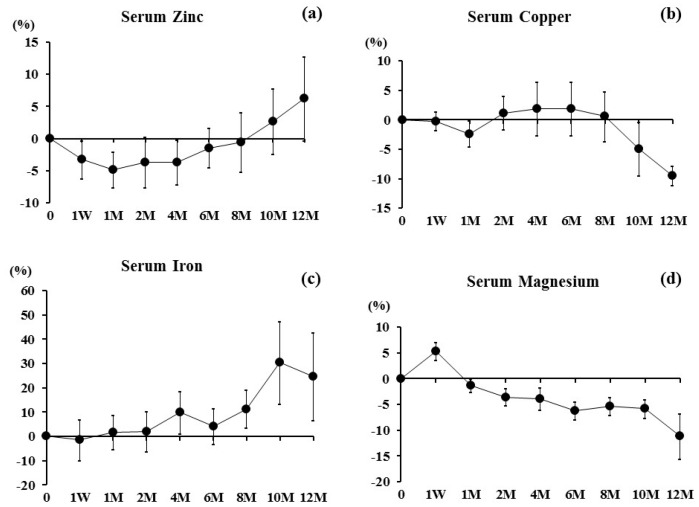
Percent changes in (**a**) Serum Zinc, (**b**) Serum Copper, (**c**) Serum Iron, and (**d**) Serum Magnesium were examined at one week and 1, 2, 4, 6, 8, 10, and 12 months after denosumab treatment. Results are expressed as the mean ± SE.

Baseline values of serum Zn, Cu, Fe, and Mg are shown in Table 1. Serum Zn tended to decrease at one week and one month, returned to the baseline values up to eight months, and further increased at 10 and 12 months (Figure 1a). Serum Cu maintained during zero to eight months, then decreased at 10 and 12 months (Figure 1b). Serum Fe gradually increased after four months (Figure 1c). Serum Mg sharply increased at one week, then decreased further (Figure 1d).

**Figure 2:**
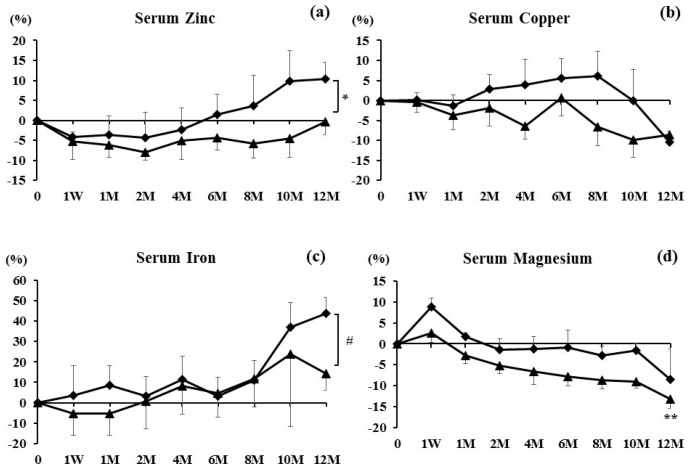
Percent changes in (**a**) Serum Zinc, (**b**) Serum Copper, (**c**) Serum Iron, and (**d**) Serum Magnesium were examined at one week and 1, 2, 4, 6, 8, 10, and 12 months after denosumab treatment. Results are expressed as the mean ± SE. Diamond shapes indicate the bisphosphonate (BP) group and triangles indicate the teriparatide (PTH) group. * *p* < 0.05 and *p* < 0.1, significant tendency between the groups at 12 months. # *p* < 0.05, significant difference between the groups at 12 months. ** *p <* 0.01, significant difference at one week or 1, 2, 4, 6, 8, 10, and 12 months compared with pretreatment.

Baseline values of serum Zn, Cu, Fe, and Mg in the PTH group and BP group are shown in Table 2. Serum Zn values decreased during the study period below the baseline values in the PTH group, while they decreased during one week to four months, returned to the baseline levels up to six months, and further increased at 10 and 12 months in the BP group. There was a statistical tendency between the groups at 12 months (*p* = 0.0603) (Figure 2a). Serum Cu values tended to decrease during the period in the PTH group, while they increased during two to eight months and thereafter decreased in the BP group (Figure 2b). Serum Fe values tended to decrease during one week to one month, then increased gradually in the PTH group, while they increased during the treatment period in the BP group. There was a significant difference between the groups at 12 months (Figure 2c) (*p* < 0.05). Serum Mg increased at one week, then decreased further in both groups. Furthermore, the Mg values were significantly decreased at 12 months in the PTH group, compared with those before treatment (*p* < 0.01). There was no significant difference between the groups (Figure 2d).

**Figure 3:**
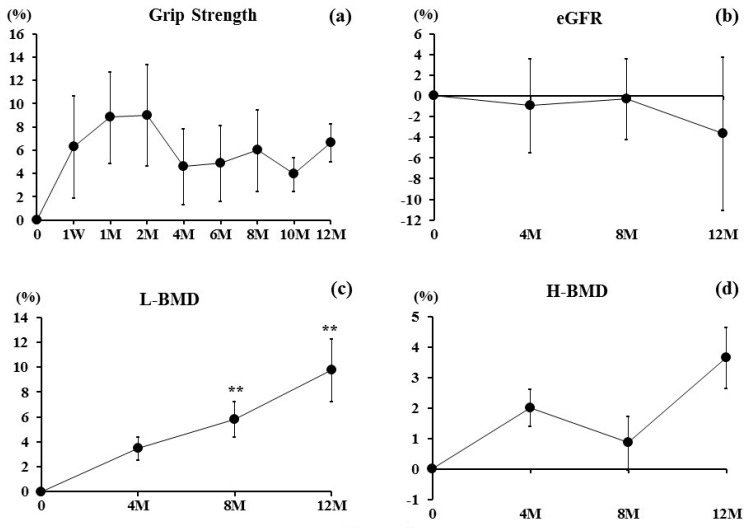
Percent changes in (**a**) Grip Strength and (**b**) Estimated Glomerular Filtration Rate (eGFR) were examined at one week and 1, 2, 4, 6, 8, 10, and 12 months after denosumab treatment. Percentage changes in bone mineral density (BMD) of (**c**) L1 to L4 lumbar vertebrae (L-BMD) and (**d**) bilateral total hips (H-BMD) at the first visit and at 4, 8, and 12 months. Results are expressed as the mean ± SE. ** *p <* 0.01, significant difference at one week or 1, 2, 4, 6, 8, 10, and 12 months compared with pre-treatment.

Grip strength increased for two months, then slightly decreased at four months, maintained 4 to 12 months (Figure 3a). eGFR almost maintained for zero to eight months, then slightly decreased at 12 months (Figure 3b). L-BMD values significantly increased at eight (5.8%) (*p* < 0.01) and 12 months (9.8%) (*p* < 0.01) (Figure 3c). H-BMD increased, but not significantly, during the period (at 12 months: 3.7%) (Figure 3d).

## 4. Discussion

We examined how serum minerals changed by anti-resorption drug denosumab treatment in post-menopausal osteoporotic Japanese patients. This study showed that at later phases of denosumab therapy, Zn and Fe tended to increase while Cu and Mg tended to decrease. Thus, denosumab might improve Zn and Fe metabolism, and it is better to obtain Mg supplementation during the therapy.

This study showed that the percent changes of serum Zn decreased during one week to six months; however, at 12 months, levels showed a 6.2% increase compared with those before treatment. Zn values were lower in the osteoporotic patients than those in the healthy people [7]. Furthermore, in osteoporotic patients, Zn losses likely occur from urinary tracts [15]. Relea et al. [16] reported that urine clearance of Zn in postmenopausal women was higher than that in premenopausal women. Steidl et al. [17] found that Zn levels in the serum were lower among the patients with postmenopausal osteoporosis than those in controls. Note that Gur et al. [15] examined the effects of alendronate (ALN) therapy on biochemical markers of bone remodeling and urinary Zn excretion over a six-month period. They reported that urinary Zn was increased after ALN therapy in post-menopausal women [15]. Furthermore, Holloway and Togari et al. previously reported that Zn stimulates osteoblasts and inhibits osteoclasts in vitro [18,19], suggesting that Zn enhances bone formation and suppresses bone resorption. Furthermore, there have been several reports that serum Zn is positively associated with BMD [20]. In addition, this study showed that there was a statistical tendency between the PTH and BP groups at 12 months (*p* = 0.0603) (Figure 2a). These findings suggest that (1) Zn is a key mineral for bone metabolism, (2) anti-resorption therapies potentially inhibit the urinary excretion of Zn and increase the serum Zn, and (3) combination therapy with denosumab and PTH might not be recommended with respect to the Zn metabolism during denosumab therapy. Thus, it is presumable that one of the mechanisms by which anti-resorption therapies increase BMD could be the increase of serum Zn.

Serum Cu decreased at 12 months. Cu is associated with decreased bone turnover, and suppresses both osteoblast and osteoclastic activity [21]. Furthermore, it is known that Cu is antagonized by Zn within the human body [22]. Thus, the reason why serum Cu decreased at 12 months might have been caused by the increase of serum Zn at 12 months after treatment with denosumab. On the other hand, Odabasi et al. described that Cu cannot be related to the increase of BMD [23]. Based on our results and a previous report [23], it is considerable that (1) Cu might be negatively associated with the changes of Zn, since serum Cu values were decreased while serum Zn values were increased at 12 months during denosumab therapy; (2) it might be better that Cu values are low at the time of osteoporotic treatment, since Cu could suppress bone metabolism.

Fe loading induced changes in bone composition, trabecular and cortical thinning of bone, as well as bone resorption [24]. Fe is essential for vitamin D metabolism [25]. Reports from animal studies have shown the relationship between dietary Fe restriction and bone health and found that severe nutritional Fe restriction has a significant impact on bone, affecting BMD, bone mineral content, and femur strength. Decreases in bone formation and/or increases in bone resorption markers were found in several studies [26,27,28]. Thus, Fe is generally an important mineral for bone metabolism. In this study, Fe increased gradually after the treatment, and the BP group had significantly increased Fe values compared with those in the PTH group at 12 months. Thus, it is suggested that the increase of Fe might have an effect on the increase of BMD after denosumab treatment—especially without PTH combination treatment.

Mg is another important essential element that effects matrix and mineral metabolism in bone [29]. Slovik et al. reported that PTH significantly lowered the serum Mg levels, but did not significantly lower its urinary secretion [30]. This study showed that serum Mg increased at one week, then gradually decreased for 12 months. In this study, 11 in 21 patients used PTH. Compared to the BP group, the PTH group had greatly decreased serum Mg level (Figure 2d). These findings suggest that serum Mg decreased at 12 months, potentially due to the PTH therapy. In Japan, after approval of denosumab use, calcium and vitamin D have been recommended to prevent hypocalcemia in osteoporosis treatment using denosumab. Thus, Denotas^®^ Chewable (calcium and vitamin D supplementation) has recently been approved during denosumab treatment. Ebina et al. have previously reported that denosumab with ALF combination therapy may be more effective than denosumab with Denotas^®^ Chewable combination therapy in terms of increasing BMD [31]. Thus, in this study, we prescribed ALF instead of Denotas^®^ Chewable supplementation; however, it is recommended to use Denotas^®^ Chewable instead of active vitamin D supplementation since Denotas^®^ Chewable also includes Mg, and denosumab might have decreased the serum Mg level when denosumab is used for a long time. In addition, Veronese et al. have very recently reported that dietary Mg intake has a protective effect for and lower risk of future osteoporotic fractures [32]. Collectively, a prospective study will be required to see whether or not Mg supplementation affects bone metabolism and fracture occurrence in a large cohort study.

There are several limitations to this study. First, as this study was based on a real-world setting, the patients were collected retrospectively. Second, this study had a small sample size; thus, it is likely that the results would change if the case number was higher. Third, since this study did not have a control group, we could not clarify the association between osteoporosis and bone-related minerals in the general population.

Our findings indicate that those minerals play key roles in bone metabolic diseases, such as osteoporosis during anti-resorption drugs.

## 5. Conclusions

In this study, serum Zn and Fe tended to increase, especially at later phases, while Cu and Mg tended to decrease during middle to later phases after denosumab therapy in Japanese post-menopausal patients. BMD increased, potentially due to the increase of Zn and Fe and the decrease of Cu, and Mg supplementation is better to take during long-term denosumab therapy.

## Figures and Tables

**Table 1:** Baseline values prior to treatment (*n* = 21).

Characteristic	Overall
Zinc (μg/dL)	70.2 ± 3.2
Copper (μg/dL)	120.3 ± 4.2
Iron (μg/dL)	76.7 ± 5.5
Magnesium (μg/dL)	2.1 ± 0.05
eGFR (mL/min/1.73 m^2^)	67.1 ± 3.6
Grip strength (kg)	15.9 ± 1.3
L-BMD (g/cm^2^)	0.761 ± 0.04
H-BMD (g/cm^2^)	0.619 ± 0.02

eGFR: estimated glomerular filtration rate, BMD: bone mineral density, L-BMD: BMD at L1 to L4 levels of the posteroanterior spine, H-BMD: BMD at bilateral hips.

**Table 2:** Baseline values prior to treatment.

Characteristic	PTH Group (*n* = 11)	BP Group (*n* = 10)	*p* Value
Age	74.1 ± 2.3	73.7 ± 2.9	*p* = 0.9147
BMI (kg/cm^2^)	20.1 ± 0.4	20.3 ± 0.7	*p* = 0.8476
Zinc (μg/dL)	70.7 ± 4.3	68.7 ± 4.9	*p* = 0.6500
Copper (μg/dL)	124.0 ± 4.6	116.2 ± 7.2	*p* = 0.3763
Iron (μg/dL)	78.8 ± 4.4	73.9 ± 6.4	*p* = 0.5380
Magnesium (μg/dL)	2.1 ± 0.06	2.2 ± 0.07	*p* = 0.9616
L-BMD (g/cm^2^)	0.760 ± 0.04	0.767 ± 0.03	*p* = 0.8872
H-BMD (g/cm^2^)	0.620 ± 0.01	0.614 ± 0.02	*p* = 0.7984

BMI: body mass index, BMD: bone mineral density, L-BMD: BMD at L1 to L4 levels of the posteroanterior spine, H-BMD: BMD at bilateral hips, PTH: parathyroid hormone, BP: bisphosphonate.
